# Burrow characteristics and ecological significance of *Marmota himalayana* in the northeastern Qinghai‐Tibetan Plateau

**DOI:** 10.1002/ece3.7754

**Published:** 2021-06-15

**Authors:** Shu‐Lin Wang, Fu‐Jiang Hou

**Affiliations:** ^1^ State Key Laboratory of Grassland Agro‐Ecosystems Key Laboratory of Grassland Livestock Industry Innovation Ministry of Agriculture Lanzhou China; ^2^ College of Pastoral Agriculture Science and Technology Lanzhou University Lanzhou China

**Keywords:** alpine meadow, burrow characteristics, burrowing animals, ecological adaptability, terrains

## Abstract

Burrows provide burrowing animals with a place to hibernate, reproduce, and avoid predators and harsh weather conditions and thus have a vital impact on their survival. However, the general physical characteristics and ecological functions of *Marmota himalayana* burrows as well as whether there are differences in burrow traits under different terrains (e.g., sunny slopes, shady slopes, and flat areas) are not well understood. From July to August 2019 (warm season), we used unmanned aerial vehicles to fly at low altitudes and slow speeds to locate 131 *M*. *himalayana* burrows (45 on shaded slopes, 51 on sunny slopes, and 35 on flat areas) in the northeastern Qinghai‐Tibetan Plateau region. We then measured the physical characteristics (burrow density, entrance size, first tunnel length, volume, orientation, and plant characteristics near the burrow entrance) of these burrows on site. We found that terrain had a substantial influence on burrow density, orientation, and entrance size and on the angle of the burrow entrance; species richness had a substantial impact on path density and tunnel volume. The physical parameters of the *M. himalayana* burrows showed that they function to protect the marmots from natural enemies and bad weather, provide good drainage, and maintain a stable microclimate around the entrance. We discuss the ability of burrowing animals (e.g., *M. himalayana*) to adapt to the external environment based on their burrow characteristics.

## Graphical abstract

The physical parameters of the *M. himalayana* dens showed that they function to protect the marmots from natural enemies and bad weather, provide good drainage, and maintain a stable microclimate around the entrance. This is a result of the marmot’s adaptation to the harsh environment (cold and humidity) of the Qinghai‐Tibet Plateau.

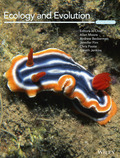

## 1 INTRODUCTION

Burrows used by burrowing animals have a vital impact on their survival. Burrows provide animals with a place to rest, hide, and hibernate and help them avoid bad weather and predators (Ross et al., 2010; Tsunoda et al., 2018). The ecological characteristics of a burrow can accurately reflect the life, behavior, and social networks of burrowing animals. For example, the yellow‐bellied marmot (*Marmota flaviventris*) in North America uses burrows that protect them from harsh environments and predators during hibernation and provide a protective shelter in summer (Svendsen, 1976). Therefore, burrows offer an excellent means of studying the ecological adaptation of burrowing animals (Ballová et al., 2019; Guo et al., 2020).

Except for plateau rabbits (*Lepus oiostolus*), *Marmota himalayana* (hereafter referred to as the marmot) is the only large rodent in the alpine meadows of the Qinghai‐Tibetan Plateau (QTP) (Zhang et al., 2019). Marmots are of great significance to the stability of the alpine meadow ecosystem. The marmot is an important part of the food chain in the grassland ecosystem, as it is important prey for large raptors, foxes, and wolves (Buyandelger et al., 2021). In addition, the burrowing and digging carried out by marmots is conducive to the circulation of organic matter in grasslands (Ballová et al., 2019). However, marmots do also cause obvious harm, as they dig grassroots and destroy turf all year round. In pastures in this region, marmot holes are the main hidden danger that causes livestock leg fractures (Chen, 1982). Each of their excavated mounds covers a large area of grassland (~2 m^2^), causing ground collapse, soil erosion, and desertification (Wang et al., Unpublished observations). Determining the adaptation mechanism of *M. himalayana* with respect to alpine meadows on the QTP in terms of its burrow characteristics could strengthen our understanding of the ecological significance of this species.

Habitat selection is a reflection of the environmental, ecological, and physiological requirements of a species (Kohji & Kenichi, 1998). When burrowing animals excavate burrows, they typically show a strong selectivity with respect to the surrounding environment. For example, marmots in the Zoige wetland, in the eastern region of the QTP, require habitats characterized by flat ground with low soil moisture content and relatively low vegetation height and density (Guo et al., 2020). The habitat choice in Tatra marmot (*Marmota marmota latirostris* Kratochvíl, 1961) is conditioned by the presence of convex geomorphic features, which are due to their structure being suitable for the construction of permanent burrows, mainly hibernacula (Ballová & Šibík, 2015). Burrows of hoary marmot (*Marmota caligata*) are predominantly in talus patches, which provide shelter from predators and weather (Karels et al., 2004). Alpine marmots (*Marmota marmota*) prefer southern and eastern exposed locations (Lenti‐Boero, 2003). Temperature and the presence of glacial and diluvial sediments are key factors influencing the distribution of *M. himalayana* (Nikol'skii & Ulak, 2006). The terrain of the QTP is extremely complex, and its influence on burrow site selection by *M. himalayana*, as well as on burrow traits, is unclear.

In this study, marmot burrows in the harsh environmental conditions of this region (low oxygen, low temperature, and high precipitation) (Zhang et al., 2019) that were present in different terrains were located, and the physical characteristics of these burrows were measured. Here we determined (a) the general physical characteristics and ecological functions of marmot burrows and (b) whether there are differences in burrow traits under different terrains. Our findings highlight the adaptation mechanism of *M. himalayana* with respect to its environment, which is of great significance for further understanding the ecological characteristics of the marmot and of other burrowing animals all over the world.

## 2 MATERIAL AND METHODS

### 2.1 Study area

The present study was undertaken at the Lanzhou University Research Station in Maqu County, Gansu Province, China (101°53′E, 33°58′N, 3,500 m a.s.l.). This area is located in the northeast of the QTP. The climate is cold and humid, with only a warm season (May to September) and cold season (October to April) (Sun et al., 2015). There is no absolute frost‐free period throughout the year. The annual average temperature is about 1.2℃, and the highest temperatures are from June to August, with an average of <12℃; the lowest temperatures (average of −10℃) are from December to February. The average annual rainfall is ~620 mm, which occurs mainly during the forage growing season (May to September). These soils are classified as Mat‐Cryic Cambisols based on previous experimental work (Sun et al., 2015), and the vegetation is characteristic of a typical alpine meadow (Yang et al., 2019). The entire study area has undulating mountains, with steep, changeable, complex, and fragmented terrain.

### 2.2 Research object

Marmots are hibernating animals. When the temperature is consistently <10℃, they will hibernate naturally for 5–6 months and will then wake up naturally when the temperature warms (Zhang et al., 2019). Marmots are family burrow social animals. Burrows are generally classified as hibernation burrows, summer‐living burrows, and temporary burrows according to their functions. Each family has a burrow group (Ballová & Šibík, 2015). The burrow group is centered on a hibernation burrow and is surrounded by several summer‐living burrows and temporary burrows (Zhang & Ma, 1984).

The natural enemies of marmots in this study area are mainly stray dogs (*Canis lupus familiaris*), Tibetan foxes (*Vulpes ferrilata*), and large raptors (*Bubo bubo* and *Buteo hemilasius*). Marmots are very cautious and often look up to observe the surrounding environment during foraging. Their area of activity is typically concentrated within 2–100 m of their burrow entrance (Yang & Xie, 1983). When they are disturbed by humans or other predators, they will sound an alarm (Blumstein & Munos, 2005), and the surrounding individuals will immediately enter a burrow after hearing the alarm (Shi, 2007; Unpublished observations, 2019). Marmots in this region begin to hibernate during mid‐October and are almost all hibernating by the end of October; they end their hibernation at the end of March or in early April (Zhang et al., 2019). During the entire warm season, except for periods with severe weather (e.g., heavy rain and/or hail), they are active outside their burrows. Generally, they leave their burrows at sunrise and return at sunset (Semenov et al., 2000).

### 2.3 Burrow location and field measurement

**FIGURE 1:**
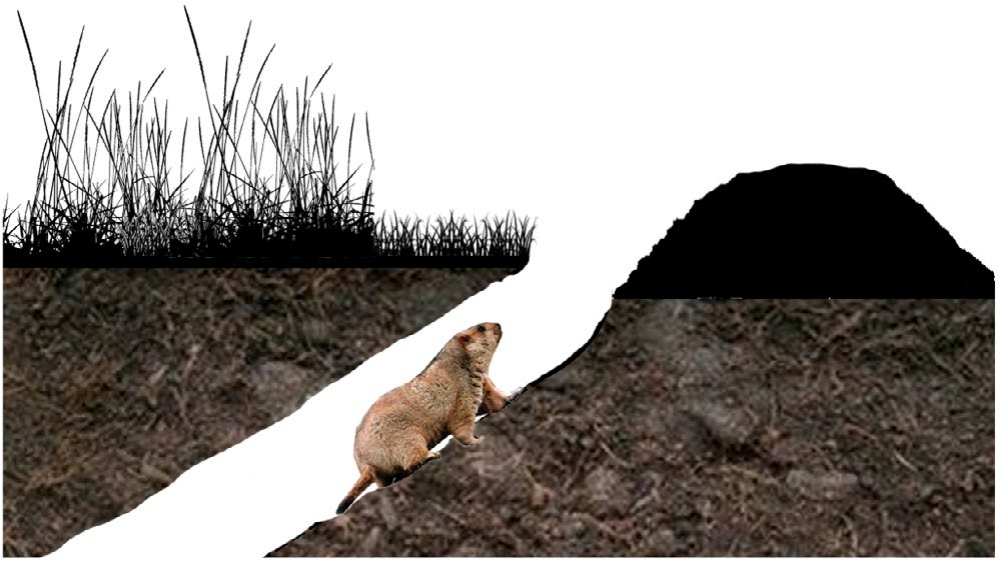
Schematic diagram of a marmot burrow

The entrance of a marmot burrow is oval in shape, with excavated soil/gravel piled up near the entrance (Figure 1), which results in a truncated cone‐shaped pile that is obviously different from the surrounding grassland (Figure S1). In addition, marmots often traverse a fixed route around the burrow entrance, trampling the grass and forming paths that are easily identified (Figure S2). Tibetan foxes occasionally are seen in the study area, and they may use burrows that have been abandoned by marmots. However, it is easy to distinguish between the burrow entrance of a fox and a marmot (Figure S1).

Marmot burrows were investigated during the summer (July to September) of 2019 by searching the study area from unmanned aerial vehicles (UAVs) flying 40–50 m above the ground at speeds of 30–50 km/h. Burrows located during aerial surveys were ground checked to verify their identity based on the presence of a large amount of marmot scat (Figure S3), footprints, trails, and the presence of an adult or cubs (Garrott et al., 1983).

During the entire study period, we investigated 131 burrows (51 on sunny slopes (facing south or west), 45 on shady slopes (facing north or east), and 35 in flat areas). To reveal the universal characteristics of *M. himalayana* burrows, we did not carefully distinguish between temporary burrows, summer‐living burrows, and hibernation burrows. We measured the following indicators of these burrows and the surrounding environment.

(1) Burrow density: We calculated the burrow density for each terrain based on the area of the surveyed sites as scanned by UAVs (Qin et al., 2020) and the number of burrows recorded.

(2) Burrow entrance size: As the burrow entrance of *M. himalayana* is oval in shape, it thus has two parameters, the long axis (*a*) and the short axis (*b*). The entrance area (S) was calculated using the following formula:

(1) $$S=\pi \times \frac{a}{2}\times \frac{b}{2}$$

(3) First tunnel length: We used a measuring tape to measure the length from the entrance to the first corner of the tunnel.

(4) Burrow volume: We used the equal volume method to measure the tunnel volume based on the pile of dirt beside each burrow. The volume of the truncated cone‐shaped pile is approximately equal to the tunnel volume:

(2) $$V\approx \frac{1}{3}\times H\times \pi \times \left(R^{2}+R\times r+r^{2}\right)$$

where V is the tunnel volume; H is the height of the mound; and *R* and *r* represent the upper and lower radius of the mound, respectively.

(5) Burrow orientation and angle of burrow entrance: We used a rangefinder (Aicevoos Z5, Shanghai, China) to measure the orientation and angle of the entrance. Here we divided the burrow orientation into eight directions: *N* (0°, at the top (12 o'clock) position), NE (1°–89°), E (90°), SE (91°–179°), S (180°), SW (181°–269°), W (270°), and NW (271°–360°).

(6) Path density near the burrow entrance: We determined the path density according to the trampled vegetation around the burrow entrance (Figure S2).

(7) Vegetation characteristics near the burrow entrance: For each burrow, to avoid any influence of the mound, we selected a quadrat (0.5 m × 0.5 m, Figure S4) in the opposite direction of the mound and 30 cm away from the burrow entrance (referred to as the near entrance quadrat). At the same time, we analyzed a control quadrat (CK) at a distance of 30 m (referred to as the activity area) away from the burrow entrance. Individual plant species (referred to as species richness) and the height of each species (referred to as the average height per species) were recorded in each quadrat. Aboveground vegetation was collected and dried to a constant weight at 65℃ before being weighed to determine aboveground biomass.

### 2.4 Statistical analyses

Data were analyzed using Statistical Package for the Social Sciences (SPSS) (version 26.0; SPSS, Inc.). Data were checked for a normal distribution using the Shapiro–Wilk test. Data for marmot burrow characteristics that were not indicated as being normally distributed were log_10_‐transformed to pursue normality and homogeneity of variances. We considered the terrain (i.e., shady slopes, sunny slopes, and flat areas) and/or distance (i.e., near the entrance and the activity area or CK) as fixed effects. One‐way analysis of variance (ANOVA) with a least significant difference (LSD) test for multiple comparisons was used to compare various indicators of marmot burrow characteristics among different terrains; for the data about plant species richness, species height, and aboveground biomass, we used a two‐way ANOVA. Figures were constructed using Origin 9.1.

Principal component analysis (PCA) was performed to study the relationship between the environmental variables (terrain and plant traits) and the burrow characteristics. PCA was performed using CANOCO version 5.0 (Šmilauer & Lepš, 2014).

## 3 RESULTS

### 3.1 Burrow density

**FIGURE 2:**
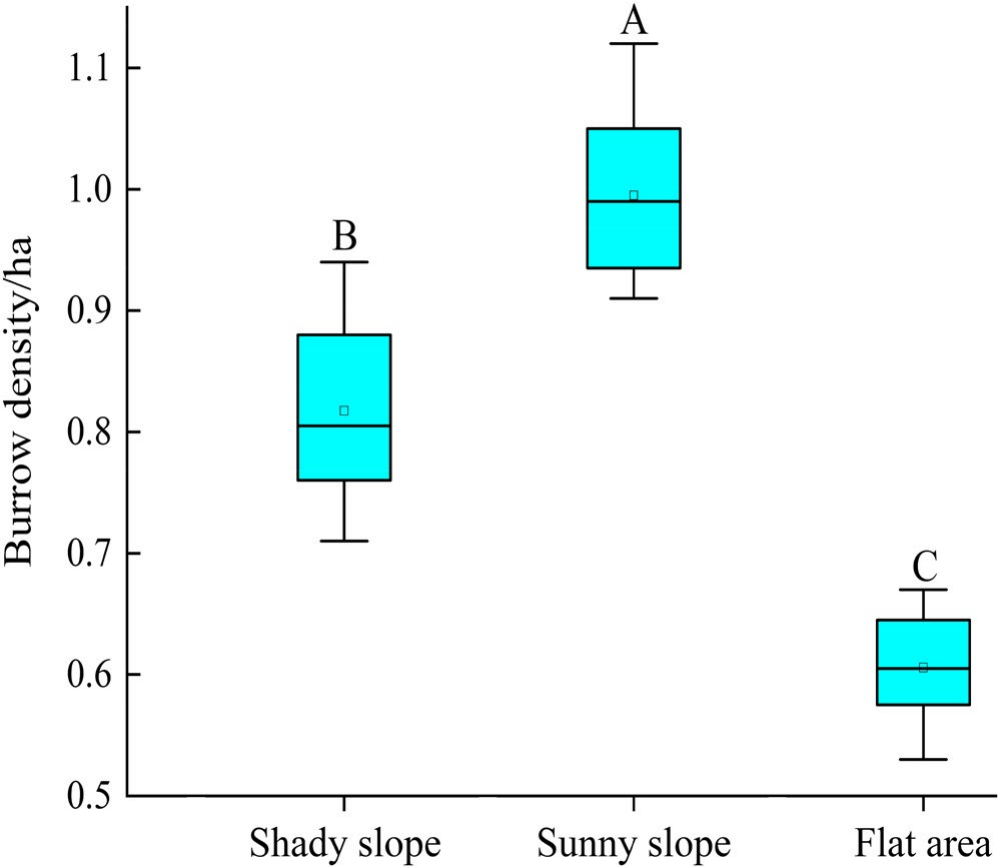
Burrow density across different terrains. Different capital letters show significant differences between different terrains (*p* < .05)

For the area searched by the UAVs, the burrow density on shady slopes, on sunny slopes, and in flat areas was 0.83, 0.97, and 0.60 burrows/ha, respectively. The burrow density on sunny slopes was significantly higher than that on shady slopes and in flat areas (*F*_2, 128_ = 3.47, *p* < .05) (Figure 2).

### 3.2 Burrow entrance size

**FIGURE 3:**
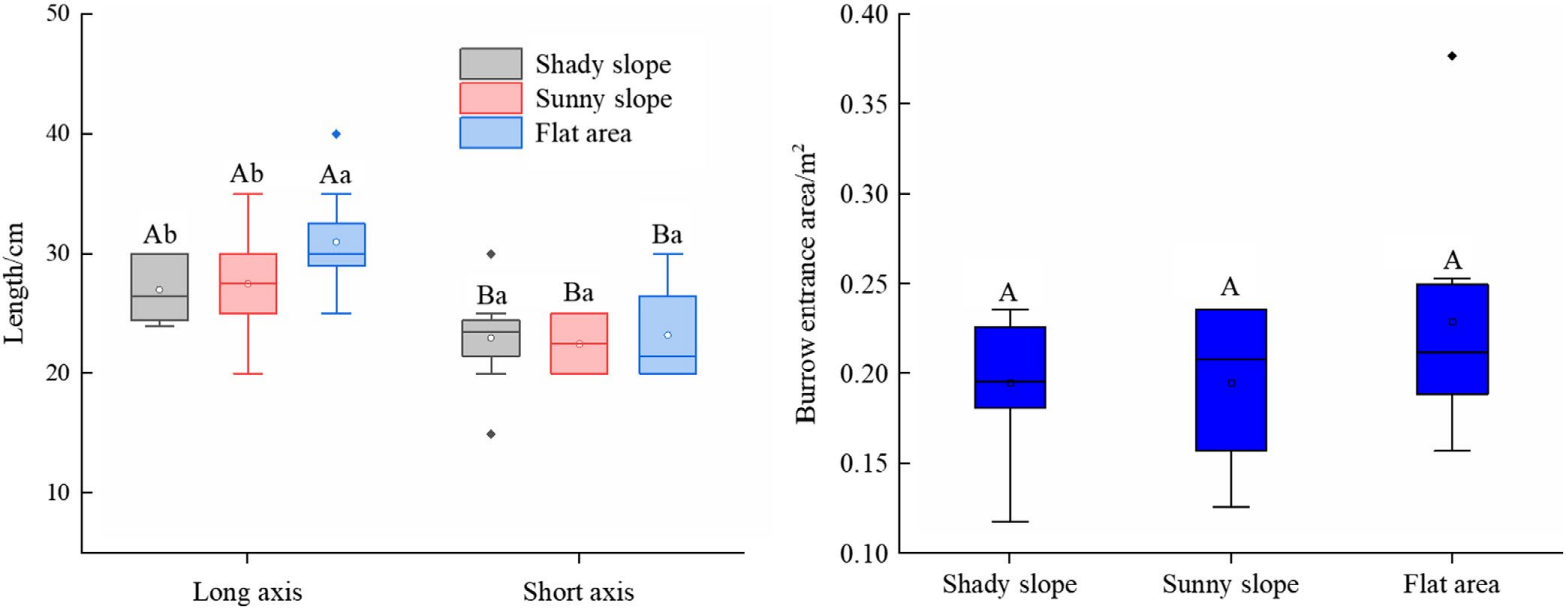
Burrow entrance shape and size across different terrains. For the burrow entrance axis measurements (left), different capital letters show significant differences between the long axis and short axis (*p* < .05); different lowercase letters show significant differences between terrains (*p* < .05) for each axis. For the burrow entrance area (right), there were no significant differences among terrains (*p* > .05). Here and throughout, the box‐and‐whisker plots show the SE data

The long axis (28.59 ± 4.32 cm) of the oval‐shaped entrances was significantly longer than the short axis (22.95 ± 3.57 cm) (*F*_1, 129_ = 3.24, *p* < .05). The average length of the long axis among entrances in flat areas (31.00 ± 4.27 cm) was significantly longer than that on sloped terrain (27.21 ± 3.71 cm) (F_1, 129_ = 3.37, *p* < .05). There was no significant difference in the length of the short axis of the entrance among different terrains (*F*_2, 128_ = 4.32, *p* = .082). In addition, there was no significant difference in the entrance area among the shady slopes (0.19 ± 0.03 m^2^), the sunny slopes (0.19 ± 0.04 m^2^), and the flat areas (0.22 ± 0.06 m^2^) (*F*_2, 128_ = 2.34, *p* = .073) (Figure 3).

### 3.3 First tunnel length

**FIGURE 4:**
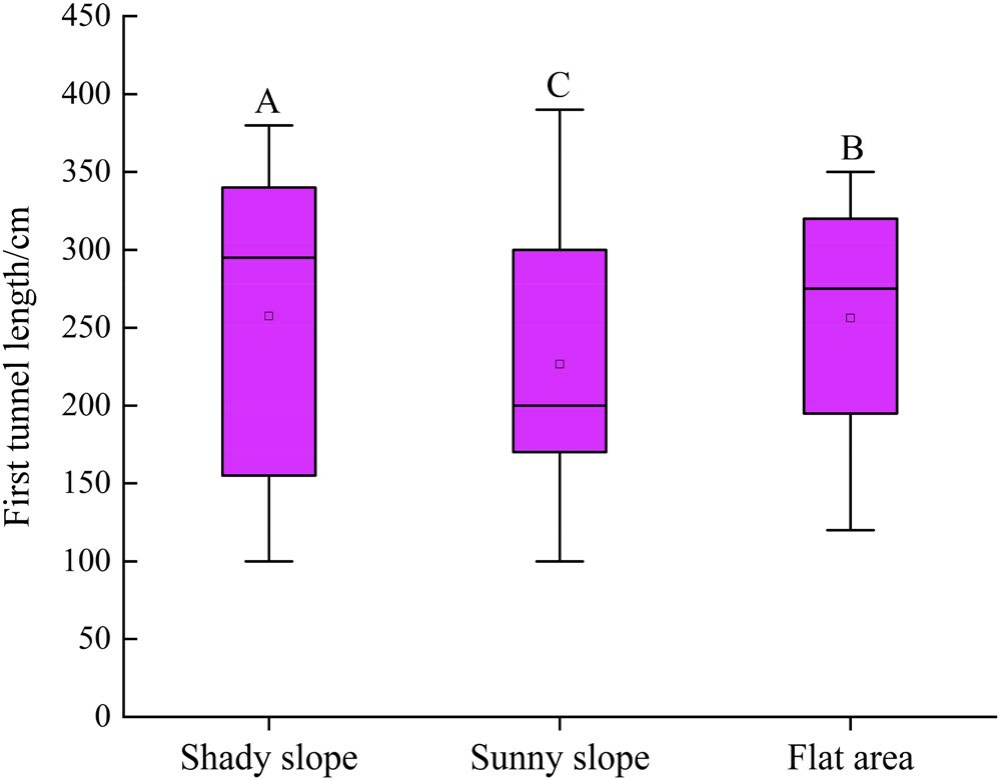
First tunnel length across different terrains. Different capital letters show significant differences between different terrains (*p* < .05)

Across all burrows, the average length of the first tunnel was 248.64 ± 23.67 cm. The first tunnel length of the burrows on the shady slopes and sunny slopes and in flat areas was 257.50 ± 101.33, 226.67 ± 93.93, and 256.25 ± 76.15 cm, respectively, and the differences among them were significant (*F*_2, 128_ = 4.37, *p* < .05) (Figure 4).

### 3.4 Tunnel volume

**FIGURE 5:**
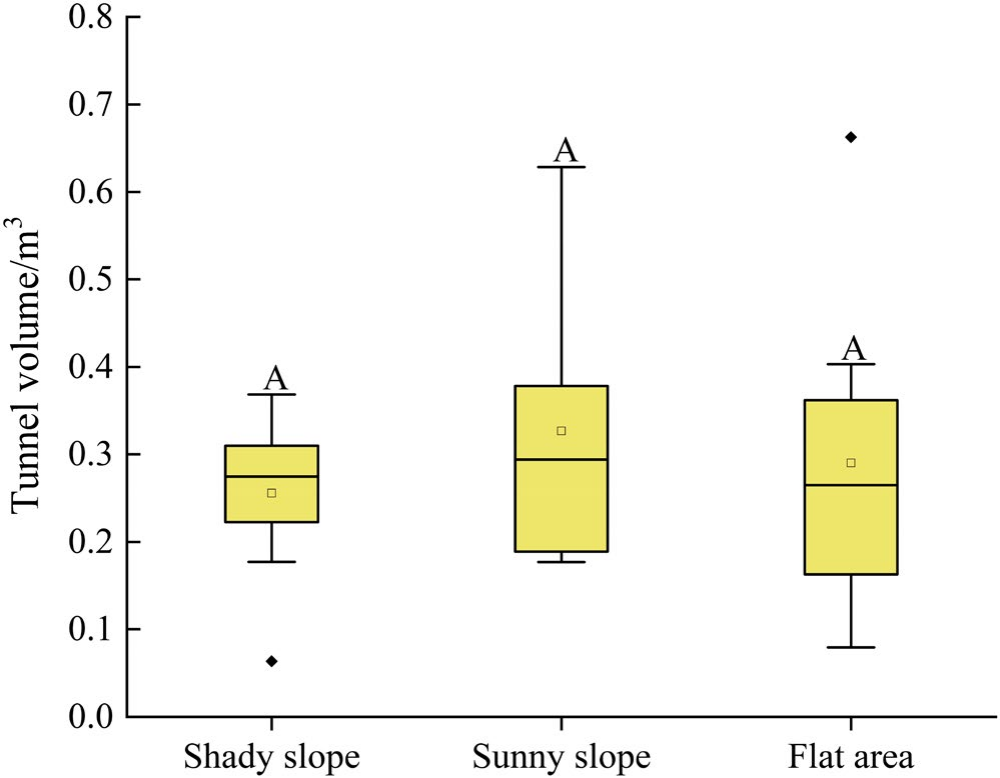
Tunnel volume across different terrains. Different capital letters show significant differences between different terrains (*p* < .05)

Tunnel volume was not significantly different among burrows on shady slopes (0.26 ± 0.08) and sunny slopes (0.32 ± 0.15) and in flat areas (0.29 ± 0.17) (*F*_2, 128_ = 3.25, *p* = .077) (Figure 5).

### 3.5 Burrow orientation

**FIGURE 6:**
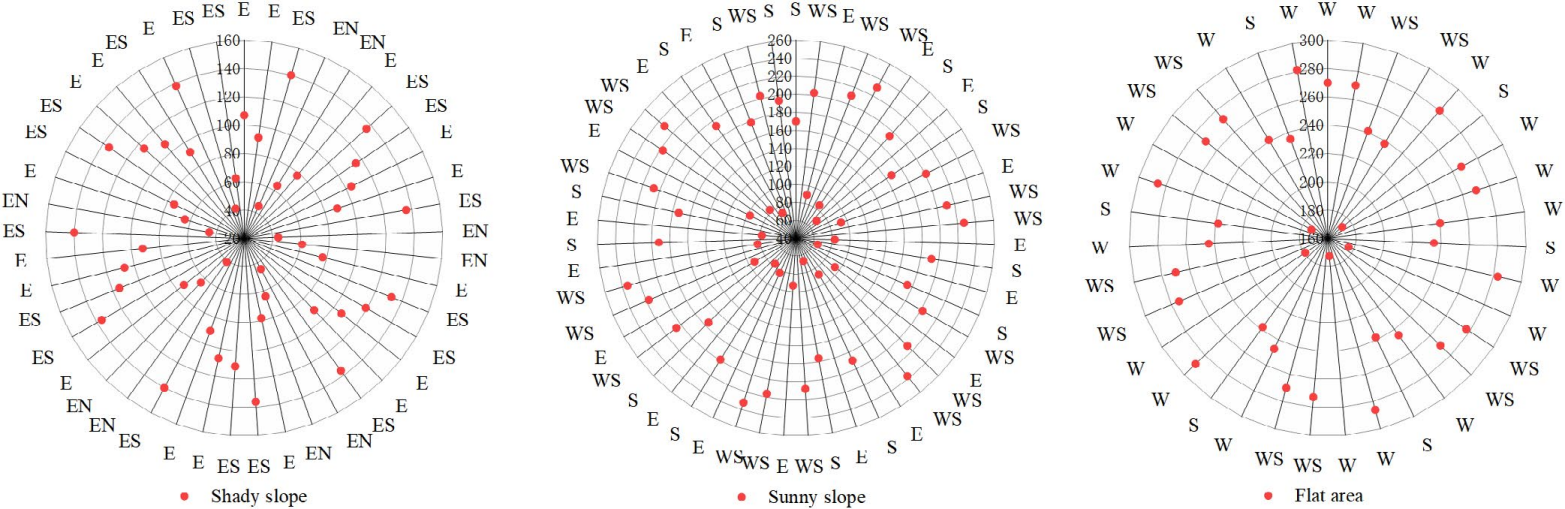
Burrow orientation across different terrains

Among the 45 dens on shady slopes, 37.50% had an east‐facing exposure, 25.00% had a northeast‐facing aspect, and 37.50% were oriented to the southeast. Among the 51 burrows on sunny slopes, 20.00% had a south‐facing exposure, 30.00% had a southwest‐facing aspect, and 50% were oriented to the west. Among the 35 burrows in flat areas, 37.50% had east‐facing exposures, 25.00% had a south‐facing aspect, and 37.50% were oriented to the southwest (Figure 6).

### 3.6 Angle of burrow entrance

**FIGURE 7:**
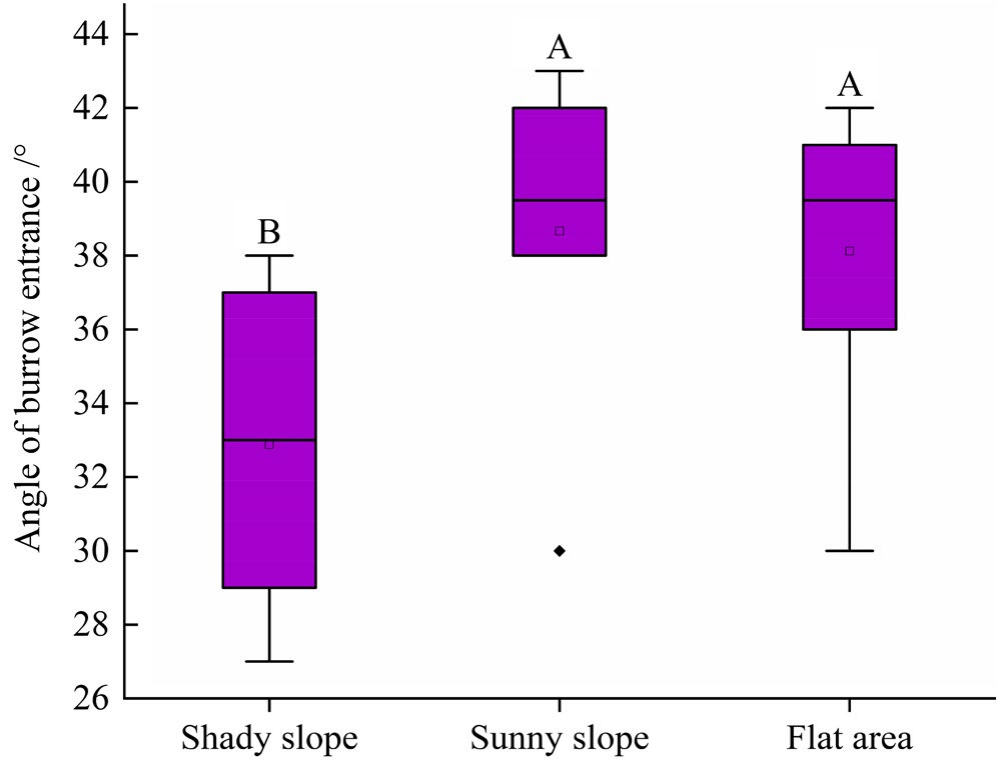
Angle of burrow entrance across different terrains. Different capital letters show significant differences between different terrains (*p* < .05)

Based on our onsite measurements, we found that the angle of the burrow entrance on shady slopes was 32.87 ± 3.98°, which was significantly lower than that on sunny slopes (38.67 ± 4.23°) and in flat areas (38.13 ± 3.92°) (*F*_2, 128_ = 1.39, *p* < .05). However, the angle of the burrow entrance was not significantly different between those on sunny slopes and in flat areas (*F*_1, 129_ = 2.37, *p* = .083) (Figure 7).

### 3.7 Path density around the burrow entrance

**FIGURE 8:**
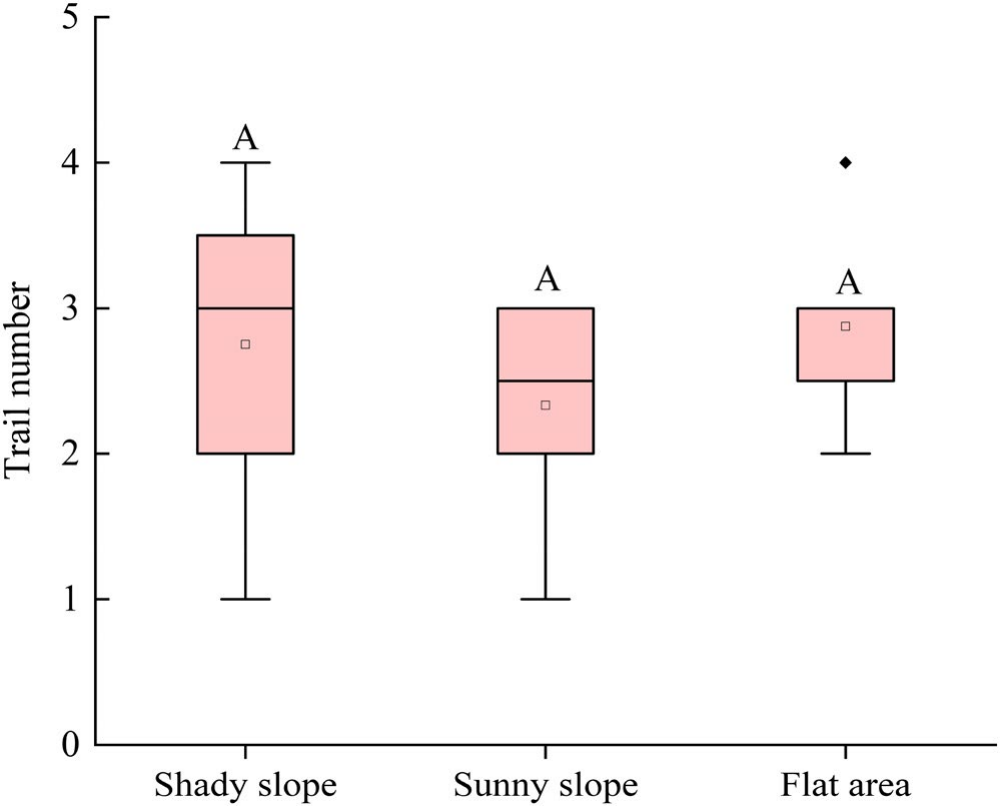
Path density across different terrains. Different capital letters show significant differences between different terrains (*p* < .05)

Path density around the burrow entrance reflects the activity intensity of *M. himalayana*. There was an average of 2.68 ± 0.82 paths per burrow. The path density for burrows on shady slopes and sunny slopes and in flat areas was 2.75 ± 0.97, 2.33 ± 0.75, and 2.88 ± 0.59, respectively; there were no significant differences in path densities (*F*_2, 128_ = 3.28, *p* = .065) (Figure 8).

### 3.8 Plant characteristics near the burrow entrance and active area

**FIGURE 9:**
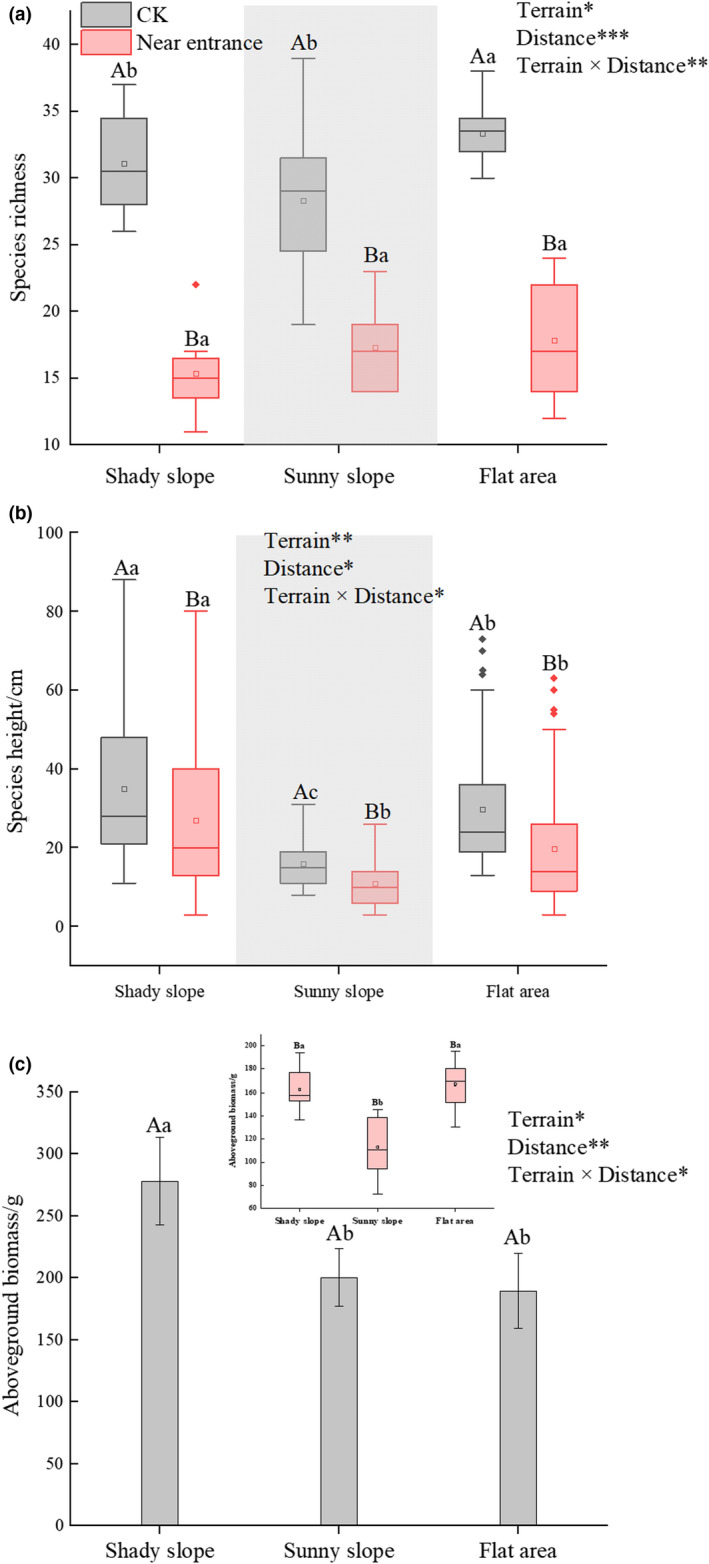
Plant characteristics near the burrow entrance and in the active area associated with each burrow. For species richness (a), species height (b), and aboveground biomass (c), different capital letters show significant differences between the quadrats near the burrow entrance relative to those in the active area (CK) (*p* < .05); different lowercase letters show significant differences between terrains (*p* < .05). Terrains consisted of shady slopes, sunny slopes, and flat areas; distances included the area near the burrow entrance and the activity area. *0.01 < *p* < .05, **0.001 < *p* < .01, ***p < .001

Species richness near the burrow entrance (16.76 ± 3.59 species) was significantly lower than that in the activity area (31.92 ± 3.43 species) across all burrows (*F*_1, 129_ = 2.73, *p* < .05). Species richness in activity areas near burrows in flat areas was significantly higher than that on sloping terrain (*F*_1, 129_ = 0.85, *p* < .05). There was no significant difference in species richness near the entrance of the burrows among the three terrains (*F*_2, 128_ = 4.41, *p* = .061) (Figure 9a).

Species height in the quadrat near the burrow entrance (20.28 ± 4.19 cm) was significantly lower than that in the activity area quadrat (28.30 ± 3.52 cm) (*F*_1, 129_ = 2.92, *p* < .05). Species height near the entrance on shady slopes (26.96 ± 4.67 cm) was significantly higher than that near the entrance on sunny slopes (11.00 ± 2.12 cm) and in flat areas (19.71 ± 3.74 cm) (*F*_2, 129_ = 3.83, *p* < .05) (Figure 9b).

Across all burrows, aboveground biomass in the activity area (222.72 ± 39.51 g) was significantly higher than that near the burrow entrance (150.77 ± 30.62 g) (*F*_1, 129_ = 2.78, *p* < .05). The aboveground biomass near the burrow entrance on sunny slopes (113.03 ± 24.17 g) was significantly lower than that on shady slopes (162.81 ± 17.96 g) and in flat areas (166.91 ± 16.70 g) (F_2, 129_ = 3.23, *p* < .05) (Figure 9c).

### 3.9 Relationship between environmental factors and burrow characteristics

**FIGURE 10:**
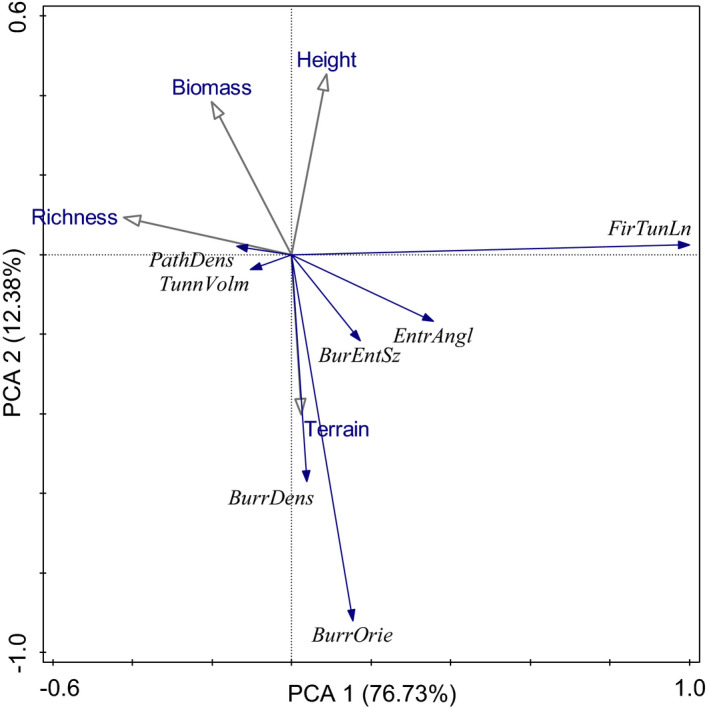
PCA of environmental variables considered in this study. Filled blue arrows indicate burrow characteristics, and open gray arrows indicate environmental factors

In a PCA of these data, principal component PC1 and PC2 explained 89.11% of the total variation in the environmental characteristics (axis 1 = 76.73%, axis 2 = 12.38%). Terrain had a substantial influence on burrow density, orientation, and entrance size and on the angle of the burrow entrance. In addition, species richness had a substantial impact on path density and tunnel volume (Figure 10).

## 4 DISCUSSION

### 4.1 Burrow site selection by marmots

The underground structure of marmot burrows is very complex, and digging a complete burrow requires a lot of work. Animals tend to use the smallest investment of energy to get the greatest return (Mcfarland, 1993). Marmots decide where to dig their burrows based on the environmental factors (i.e., the terrain, plant traits) (Zhang et al., 2019). In addition to being energy‐intensive, digging holes is also a dangerous job (as the marmots are open to predation), and these animals thus do not waste energy or carry unnecessary risk by digging more holes than are needed. Therefore, marmots exhibit strong habitat selectivity when digging holes (Guo et al., 2020).

We found that burrow density on shady and sunny slopes was higher than that in flat areas (Figure 2; Figure 10). This may be a result of topographic characteristics that directly affect the moisture and thickness of the soil (Xiao et al., 2019). A burrow must be dry and have clean underground shelters in which marmots can live (Shi, 2007). Although flat areas are conducive to digging burrows, these alpine meadows have frequent precipitation during the warm season and rainwater can easily flow into the burrow. This could explain why the burrow density is lower in flat areas. The soil layer on the shady slopes in this region is thick (~1 m) relative to that in the other two regions (e.g., ~30 cm for the sunny slopes and ~60 cm for the flat areas) (He & Li, 2016) and is therefore convenient for marmots to build their underground burrow systems. In areas where the soil is shallow and the lower layer is rocky, marmots usually use talus slopes, boulders, and reinforced rock forms with sediments (Ballová & Šibík, 2015; Karels et al., 2004). Marmots like to excavate their burrows on sunny slopes; in addition to their need for a dry and comfortable habitat, they also benefit from basking in the sun and staying warm, which is vital during periods of cold temperatures and to avoid freezing in winter after they hibernate (Shi, 2007; Türk & Arnold, 1988).

### 4.2 Burrow characteristics and functions

The shape of the burrow entrance is typically determined by the morphological structure of the inhabitant (Zhang et al., 2019). Marmots have a flattened body shape. This body shape is conducive to remaining close to the ground to avoid being discovered by predators when the marmots are active outside the burrow. Field measurements were used to determine that the long axis of the oval‐shaped entrance to these burrows was significantly longer than the short axis (Figure 3). These dimensions are larger than the burrow entrance diameter (27 and 19 cm for the long and short axis, respectively) of the steppe marmot (*Marmota bobak* Müll.) that inhabits the Ukraine (Nikol'Skii & Savchenko, 2002), as the average body weight of *M. himalayana* (5.5 kg) is larger than that of steppe marmots (3.85 kg). The shape and size of the burrow entrance facilitate the rapid entry of the marmot while preventing predators from entering (Rodrick & Mathews, 1999). We found that there was no significant difference in the area of the burrow entrance among different terrains, which suggests that the size of the burrow entrance helps to maintain a stable temperature inside the burrow (Nikol'Skii & Savchenko, 2002), which is an adaptive strategy used by marmots to protect themselves and to reduce the possible impact (i.e., sudden drop in temperature and/or rain) of the external environment (Jia et al., 1991). We also found that burrows in flat areas had a longer long axis relative to the long axis of burrows on slopes. This may be due to the wider field of vision that marmots have access to on slopes; thus, a wider burrow entrance in flat areas makes it easier for a marmot hiding inside the burrow to observe potential predators.

We found that the average first tunnel length was 248.64 cm (Figure 4), which is longer than that (127.3 cm) of burrows in other parts of the QTP (Zhang et al., 2019). This difference is most likely due to the different geographical and climatic conditions across different regions. The first tunnel is deep and long, which can increase the infiltration distance of rainwater after entering the tunnel, thereby minimizing the accumulation of water deeper within the burrow. This is especially important in rainy areas (e.g., the northeastern region of the QTP). In addition, the shortest length of the first tunnel in this study was 100 cm, which is greater than the average body length of *M. himalayana* (55 cm) and thus ensures that they can enter the burrow quickly to avoid predators (Zhang et al., 2019).

Tunnel volume reflects the internal structural traits of the marmot burrow. We found that on average the burrow volume was 0.29 m^3^, slightly larger than the hibernation chamber (0.23 m^3^) in other parts of the QTP (Wang, 1992), but there was no significant difference in the burrow volume among the different terrains analyzed here (Figure 5). The underground burrow system has a complex internal structure and multiple nests, which are places for marmots to breed, hibernate, and store food (hay) (Wang, 1992). The truncated cone‐shape mound near the burrow entrance represents an observatory for the resident marmot(s). They often stand on the mound to observe the environment around the burrow entrance (sometimes for >1 hr), and they become active around the entrance only after confirming that there is no potential danger (Yang & Xie, 1983).

Although the aspect of a burrow may confer certain advantages, the direction of the burrow opening may be related to site‐specific conditions such as vegetation, drainage, or climate (Rodrick & Mathews, 1999). Among the 131 burrows surveyed in this study, there were no burrows oriented toward the north, and only a few (9.09%) burrows located on shady slopes displayed a northeast aspect (Figure 6). Danilov (1961) suggested that burrows oriented southward have a more favorable microclimate because of the protection from the prevailing northeasterly winds. Consistent with this, alpine marmots prefer south‐oriented slopes due to their better conditions for hibernation (Shi, 2007; Türk & Arnold, 1988). Chesemore (1969) found that most entrances of arctic fox dens had a southerly, easterly, or westerly orientation, possibly indicating a preference for a warmer exposure. We did not, however, assess any microclimatic variables of the burrows in this study.

The angle between the tunnel and the ground has an important influence on the structural stability of the burrow (Chen, 1982). We found that on average, the angle of the burrow entrance was 36 ± 4.82° (Figure 7), which is smaller than that of a previous study (45°) (Zhang et al., 2019). A smaller angle of the burrow entrance results in structural instability and makes it easier for the burrow to collapse. In contrast, a larger angle makes it difficult to dig the burrow, and the excavated soil can easily backfill the tunnel; in addition, rainwater easily collects in the burrow. Thus, the moderate angle noted here is the result of a trade‐off between structural stability and better drainage (Wang & Wang, 2006).

### 4.3 Plant characteristics near the burrow entrance

Marmots are herbivores and forage mainly on the leaves and stems of Cyperaceae, Poaceae, and the flowers of Leguminosae species (Garin et al., 2008). Vegetation characteristics (richness, height, and biomass) have an important influence on the burrow‐selecting behavior of marmots (Li et al., 2017). Marmots are herbivores and forage mainly on the leaves and stems of Cyperaceae, Gramineae, and the flowers of legume species. Therefore, those species (e.g., *Kobresia pygmaea*, *Elymus nutans,* and *Thermopsis lanceolata*) are the most numerous and most common species of plants around the burrows. We found that species richness in marmot activity areas was significantly higher than that near the burrow entrance (Figure 9). The active area for *M. himalayana* is mainly concentrated within 2–100 m near the burrow entrance (Yang & Xie, 1983; Wang et al., Unpublished observations). Plants in the active area provide food and water for the marmots, as well as bedding for their nests in winter. Marmots like to feed on succulent and highly nutritious forage and can get enough water from their forage without additional water (Shi, 2007). Generally, marmots like to excavate burrows on sloped terrain, but we found that there were also some burrows in flat areas (0.60 burrows/ha), and the species richness in those areas was significantly higher than that on sloped terrain. High species richness may be the reason why marmots are attracted to these places to dig burrows (Shi, 2007).

Plant height also has a significant influence on the activities of marmots (Li et al., 2017). We found that plant height within the activity area was significantly higher than that near the burrow entrance (Figure 9). The low plants near the burrow entrance allow marmots to hide in their tunnels while observing the outside environment (Shi, 2007), thus ensuring that there is no danger before they leave their burrows. In addition, marmots often stand and observe their surroundings when feeding, and the exposed burrow entrance serves as a marker, which helps them return to their burrow quickly when danger is detected (Zhang et al., 2019).

Because marmots are active only near the entrance to their burrows, the availability of food in those areas directly affects their burrow‐selecting behavior and quality of life (Bel et al., 1995). We found that the aboveground biomass in the active area was significantly higher than that near the burrow entrance (Figure 9). Abundant food sources ensure that these marmots can reserve enough energy before hibernation to prepare for their ~6‐month hibernation period (October to April).

## 5 CONCLUSIONS

Terrain had a substantial influence on burrow density, orientation, and entrance size and on the angle of the burrow entrance; species richness had a substantial impact on path density and tunnel volume. The physical parameters of the *M. himalayana* burrows showed that they function to protect the marmots from natural enemies and bad weather, provide good drainage, and maintain a stable microclimate around the entrance. Den characteristics of *M. himalayana* are the result of adaptation to the harsh environment of the QTP. In addition, this study also provides a theoretical basis for the mechanism by which *M. himalayana* as well as other burrowing animal adapted to its environment.

## CONFLICT OF INTEREST

None declared.

## AUTHOR CONTRIBUTIONS

**Shulin Wang:** Data curation (lead); Investigation (lead). **Fujiang Hou:** Funding acquisition (supporting).

## Supporting information

App S1

## Data Availability

Data are available through Figshare (https://doi.org/10.6084/m9.figshare.13365329).
