# Supplementary material for: Burrow characteristics and ecological significance of Marmota himalayana in the northeastern Qinghai‐Tibetan Plateau
Source: Ecol Evol. 2021 Jun 15;11(13):9100–9. doi: 10.1002/ece3.7754 (PMC8258228; doi:10.1002/ece3.7754)
Supplement: Supplementary file 1 — App S1 [file ECE3-11-9100-s001.docx]

**Appendix**


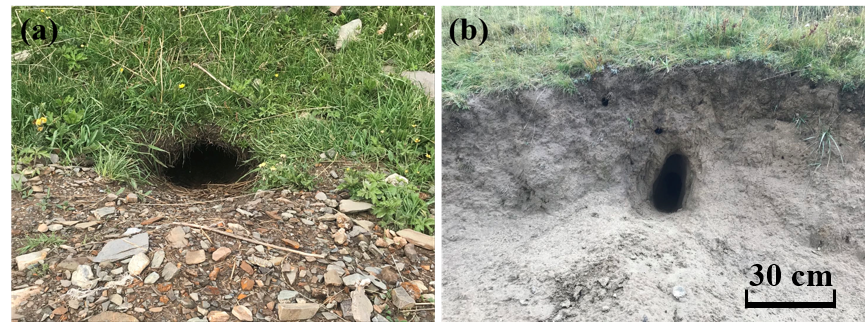


**Fig. S1.** Marmot den (a) and Tibetan fox den (b).


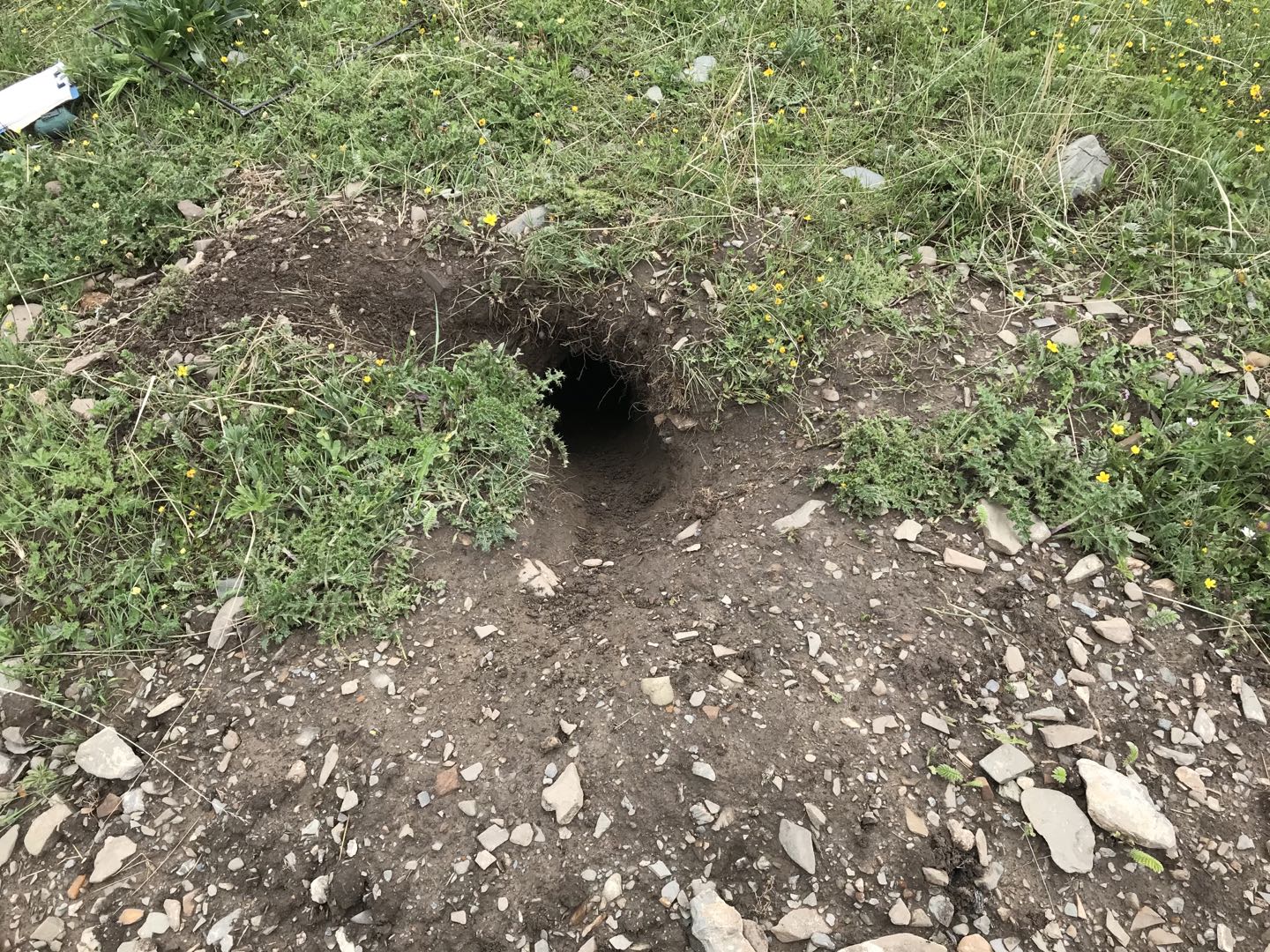


**Fig. S2.** Paths (marked by white arrows) near the den entrance.


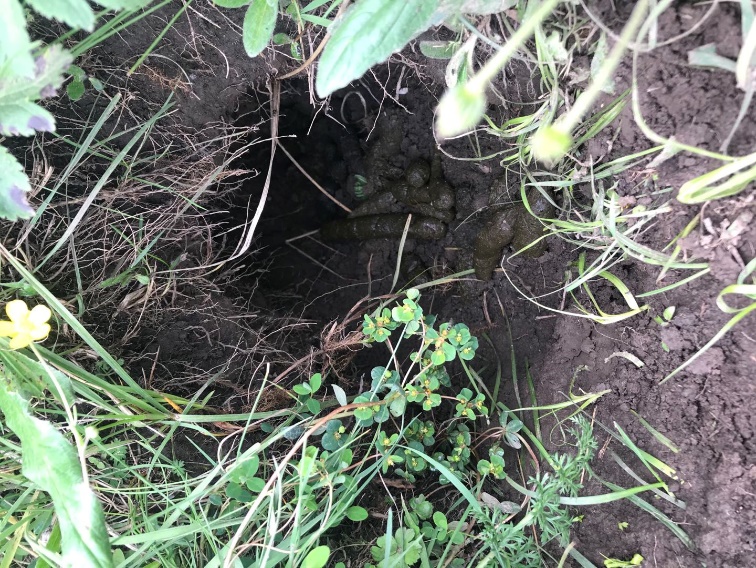


**Fig. S3.** Fresh marmot scat.


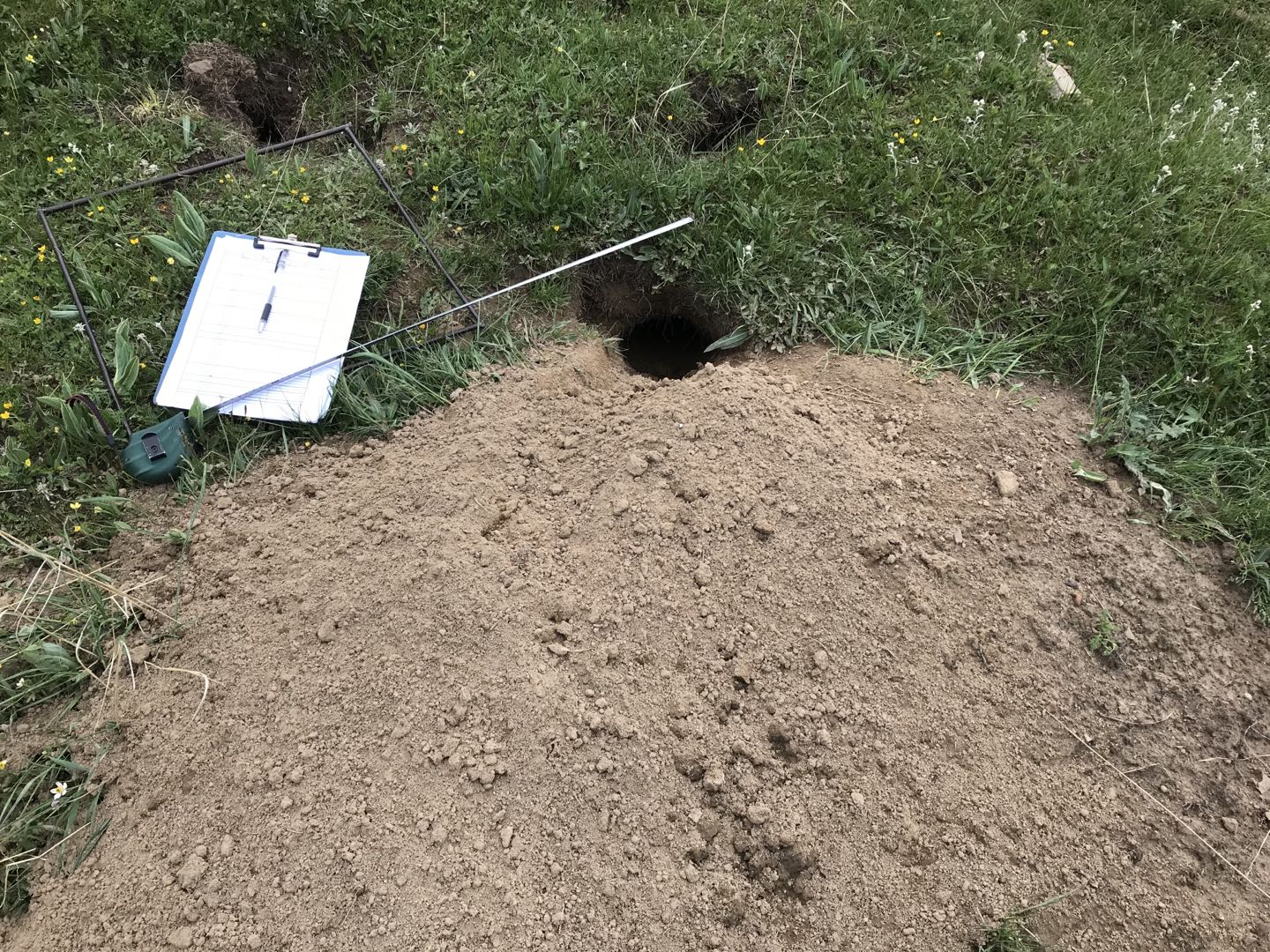


**Fig. S4.** Vegetation characteristics near the burrow entrance (quadrat size of 0.5 m × 0.5 m).
